# Comparative analysis of enzymatic profiles and biofilm formation in clinical and environmental *Candida kefyr* isolates

**DOI:** 10.1111/1758-2229.13282

**Published:** 2024-06-23

**Authors:** Hasti Nouraei, Samira Zare, Maryam Nemati, Neda Amirzadeh, Marjan Motamedi, Shafigheh Shabanzadeh, Kamiar Zomorodian, Keyvan Pakshir

**Affiliations:** ^1^ Department of Parasitology and Mycology, School of Medicine Shiraz University of Medical Sciences Shiraz Iran; ^2^ Basic Sciences in Infectious Diseases Research Center, School of Medicine Shiraz University of Medical Sciences Shiraz Iran

## Abstract

The global landscape of *Candida* infections has seen a significant shift. Previously, *Candida albicans* was the predominant species. However, there has been an emergence of non‐*albicans Candida* species, which are often less susceptible to antifungal treatment. *Candida kefyr*, in particular, has been increasingly associated with infections. This study aimed to investigate the profiles of enzymatic activity and biofilm formation in both clinical and non‐clinical isolates of *C. kefyr*. A total of 66 *C. kefyr* isolates were analysed. The activities of proteinase and phospholipase were assessed using bovine serum albumin and egg yolk agar, respectively. Haemolysin, caseinolytic and esterase activities were evaluated using specific methods. Biofilm formation was investigated using crystal violet staining. The findings indicated that biofilm and proteinase activity were detected in 81.8% and 93.9% of all the isolates, respectively. Haemolysin activity was observed with the highest occurrence (95.5%) among normal microbiota isolates. Esterase activity was predominantly identified in dairy samples and was absent in hospital samples. Caseinase production was found with the highest occurrence (18.2%) in normal microbiota and hospital samples. Phospholipase activity was limited, found in only 3% of all the isolates. These findings reveal variations in enzyme activity between clinical and non‐clinical *C. kefyr* isolates. This sheds light on their pathogenic potential and has implications for therapeutic strategies.

## Graphical abstract

This study investigates the enzymatic profiles and biofilm formation in clinical and environmental *Candida kefyr* isolates. Findings reveal significant biofilm, proteinase and haemolysin activities across isolates. Esterase activity was mainly found in dairy samples, while caseinase production was prevalent in normal microbiota and hospital samples. Phospholipase activity was minimal. The study highlights the pathogenic potential of *C. kefyr* and its implications for therapeutic strategies.

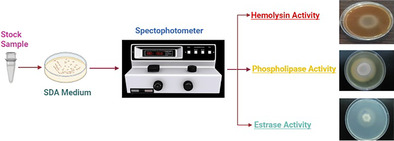

## INTRODUCTION

Over the past few years, there has been a significant increase in the occurrence and frequency of invasive fungal infections, such as candidiasis. The increase in numbers can be attributed to the usage of corticosteroids and antibacterial drugs in the field of clinical medicine (Ciurea et al., 2020; Talapko et al., 2021). *Candida* is a known factor in the development of fungal yeast infections. The clinical signs and symptoms of candidiasis are highly varied, and this condition can present in various forms, ranging from surface‐level afflictions to systemic disease (Mukaremera et al., 2017).

The international community has observed a notable shift in the incidence rates of *Candida* infections. This shift is characterised by a gradual change from the predominant incidence of *Candida albicans* to a more frequent occurrence of non‐*albicans Candida* species (Giacobbe et al., 2020).

This change can be attributed to the widespread use of antifungal prophylaxis, which has led to infections by *Candida* species that exhibit reduced susceptibility to existing treatments (Bhattacharya et al., 2020). More recently, the emergence of Candidiasis caused by less common species, such as *Candida kefyr*, has introduced a new health challenge, particularly for patients in hospital settings (Ahmad et al., 2020). *C. kefyr* (formerly *Candida pseudotropicalis*) is a yeast with its teleomorph currently recognised as *Kluyveromyces marxianus*. Individuals with compromised immune systems, especially those suffering from haematological malignancies, have reported cases of *C. kefyr*. This organism is implicated in causing a variety of conditions, including urinary tract infections, endocarditis, fungal keratitis and onychomycosis (Nurdin et al., 2021). Various studies have identified *Candida* species, including *kefyr*, residing in dairy products like milk and cheese. Furthermore, *C. kefyr* has been identified as a source of mastitis in ruminant animals like cows and goats, which impacts both the milk's quality and quantity, and it has also been detected in the milk of asymptomatic goats (Zaragoza et al., 2021). The consumption of dairy products that contain *C. kefyr* may facilitate the transmission of these organisms to individuals who are at increased risk, particularly hospitalised patients and those with underlying medical conditions (Nurdin et al., 2021). The presence of this species in dairy items enhances its ability to establish itself within the gastrointestinal tract, thereby elevating the probability of colonisation events (Wawron et al., 2011). This situation underscores the importance of monitoring and potentially regulating the intake of such dairy products among vulnerable populations to mitigate the risk of infection. The development of candidiasis is influenced by important factors such as adhesion, biofilm formation, hypha formation, dimorphism and enzyme activity (McCall et al., 2019). *Candida* species have unique protein molecules that aid in attaching to both non‐biological surfaces and host cells. Through biofilm formation, this organism creates a cohesive structure that allows it to colonise and adhere to a variety of surfaces (Cavalheiro & Teixeira, 2018). The development of *Candida* biofilms has important clinical implications, as they exhibit increased resistance to antifungal therapy and can evade detection by the immune system (Nett & Andes, 2020).

*Candida* species pathogenicity is significantly influenced by the production of exoenzymes such as proteinase, phospholipase, haemolysin and esterase, which aid in the organism's invasion into host tissues. These enzymes target proteins, phospholipids, red blood cells and esters, respectively, leading to the breakdown of host cell membranes and the extracellular matrix (Sharma et al., 2017). This breakdown not only facilitates the penetration and spread of the fungus within the host but also helps it evade the host's immune responses (Al‐Dafay et al., 2021). These exoenzymes rupture cell membranes, leading to cellular lysis, inflammation, and tissue damage, which increases the symptoms and development of *C. kefyr* infections (Ahmad et al., 2020).

The enzymatic profiles of *Candida* species isolated from various patients show significant differences based on the specific disease and the site of isolation (Nouraei et al., 2020). The objective of this study was to investigate the severity of biofilm production and analyse the enzymatic activity profiles of phospholipase, protease, haemolysin, caseinase and esterase in clinical and non‐clinical *C. kefyr* species. Additionally, this study includes the first‐ever evaluation of the caseinolytic activity exhibited by these species.

## EXPERIMENTAL PROCEDURES

### Samples

A total of 66 *C. kefyr* isolates, comprising 22 clinical (leukaemia, HIV+ and diabetic patients) 22 non‐clinical, representing normal microbiota (vagina and mouth) and 22 environmental samples obtained from dairy products (cheese, milk and buttermilk), underwent evaluation. The identification of these samples was carried out using molecular methods in prior studies, and they have been preserved in the mycology department of the Shiraz University of Medical Sciences as stocks.

### Enzyme activities

#### Proteinase and phospholipase activity

The measurement of proteinase activity utilised a bovine serum albumin medium, as described by Dagdevire et al. (2005). Six‐millimetre filter paper disks are immersed in a solution containing 10^7^ cells/mL and then placed on the plates. For 6 days, the plates were incubated at 35°C, and the opacity of the agar surrounding the disks was measured. Phospholipase activity, according to Price et al. (1982), was evaluated using the egg yolk agar method. To prepare the medium, 5.5 g of CaCl_2_ and 58.4 g of NaCl were added to Sabouraud dextrose agar (Merck, Germany). After centrifuging a sterile egg yolk at 5000 × g for 30 min, 20 mL of the supernatant was added to the cooled medium. After making a McFarland equivalent to 2 turbidity yeast suspension, 10 μL of this suspension was spot‐inoculated onto the plate medium and then incubated for up to 5 days at 35°C. The colonies' precipitant halo was evaluated, and the precipitation zone (Pz) for both proteinase and phospholipase was expressed as follows: Pz negative (1), Pz 1+ (0.9–0.99), Pz 2+ (0.8–0.89), Pz 3+ (0.7–0.79), and Pz 4+ (<0.7).

#### Caseinolytic and esterase activity

Caseinolytic activity was assessed using a casein agar plate comprising 20.0 g of glucose, 16.0 g of peptone, 5.0 g of yeast extract, 4.0 g of casein (obtained from Sigma‐Aldrich) and 16.0 g of agar prepared in 1000 mL of distilled water and autoclaved. To detect the casein degradation, a halo surrounding the fungal inoculum on the plates was measured (Ziccardi et al., 2015).

To evaluate esterase production, a Tween (Sigma‐Aldrich) agar plate was used. The plate was prepared by combining 1 g of peptone, 0.5 g of NaCl and 0.01 g of CaCl_2_ in 100 mL of distilled water with a pH of 7.0. After adding 1.5 g of agar, the medium was autoclaved and cooled to around 50°C. Next, 0.5 mL of autoclaved Tween was added to the medium. The hydrolysis of Tween resulted in the release of fatty acids that bound to calcium, leading to the formation of a Pz around the fungal colonies (Slifkin, 2000).

#### Haemolysin activity

The haemolytic activity assessment was carried out using a blood plate assay, following the methodology described by Luo et al. (2001), with specific modifications outlined by Linares et al. (2007). To prepare the sheep blood sabouraud dextrose agar, fresh sheep blood was mixed at a concentration of 7% v/v along with 3% w/v glucose. Subsequently, a 2 McFarland turbidity suspension was prepared. A 10 μL volume of the presence of a distinctive translucent halo around the inoculation site indicated positive haemolytic activity.

The haemolytic index, indicating the intensity of haemolysin production, was determined by dividing the diameter of the colony by the sum of the colony's diameter and the diameter of the translucent halo surrounding it. All experiments were performed in triplicate.

#### Detection of biofilm formation

Biofilm formation was conducted following the method described by Jin et al. (2003) with modifications by Melo et al. (2007). Initially, 100 μL of a standardised cell suspension containing 10^7^ cells/mL was added to flat‐bottom 96‐well microtiter plates and incubated for 1.5 h at 37°C in a shaker at 75 rpm. Eight wells on each microtiter plate without *Candida* spp. were used as controls. Then, the cell suspensions were carefully discarded, and each well was washed twice with 150 μL of phosphate‐buffered saline (PBS). Next, 100 μL of yeast nitrogen base medium (Sigma‐Aldrich) supplemented with 50 μM of glucose (d‐glucose monohydrate) was added to each well and incubated at 37°C in a shaker at 75 rpm for 66 h. Afterwards, the wells were washed twice with 150 μL of PBS and air‐dried for 45 min. Subsequently, each washed well was stained with 110 μL of a 0.4% aqueous crystal violet solution for 45 min. Finally, each well was washed four times with 350 μL of sterile distilled water and immediately destained with 200 μL of 95% ethanol. After a further 45 min, 100 μL of the destaining solution was transferred to a new well, and the amount of crystal violet stain was measured using a microtiter plate reader (BMG LABTECH) at a wavelength of 570 nm.

Biofilms were categorised into four density levels based on established cut‐off values (ODc), calculated from the mean values of negative controls (mean ODnc) plus three times the standard deviation of negative controls (3 × SDnc): ODc = mean ODnc + (3 × SDnc). The density categories are as follows:

$$\mathrm{OD}\leq {\mathrm{OD}}_{C}=\text{negative for biofilm}$$

$${\mathrm{OD}}_{C}\leq \mathrm{OD}\geq 2\times {\mathrm{OD}}_{C}=\text{mildly positive for biofilm}$$

$$2\times {\mathrm{OD}}_{C}\leq \mathrm{OD}\geq 4\times {\mathrm{OD}}_{C}=\text{moderately positive for biofilm}$$

$$4\times {\mathrm{OD}}_{C}\leq \mathrm{OD}=\text{intensely positive for biofilm}$$

Each experimental condition was repeated three times (Turan & Demirbilek, 2018).

### Statistic analysing

The statistical analyses for the enzyme tests consisted of the Chi‐square test and Fisher's exact test using the SPSS Statistics programme.

## RESULTS

### Enzymatic activity

**FIGURE 1:**
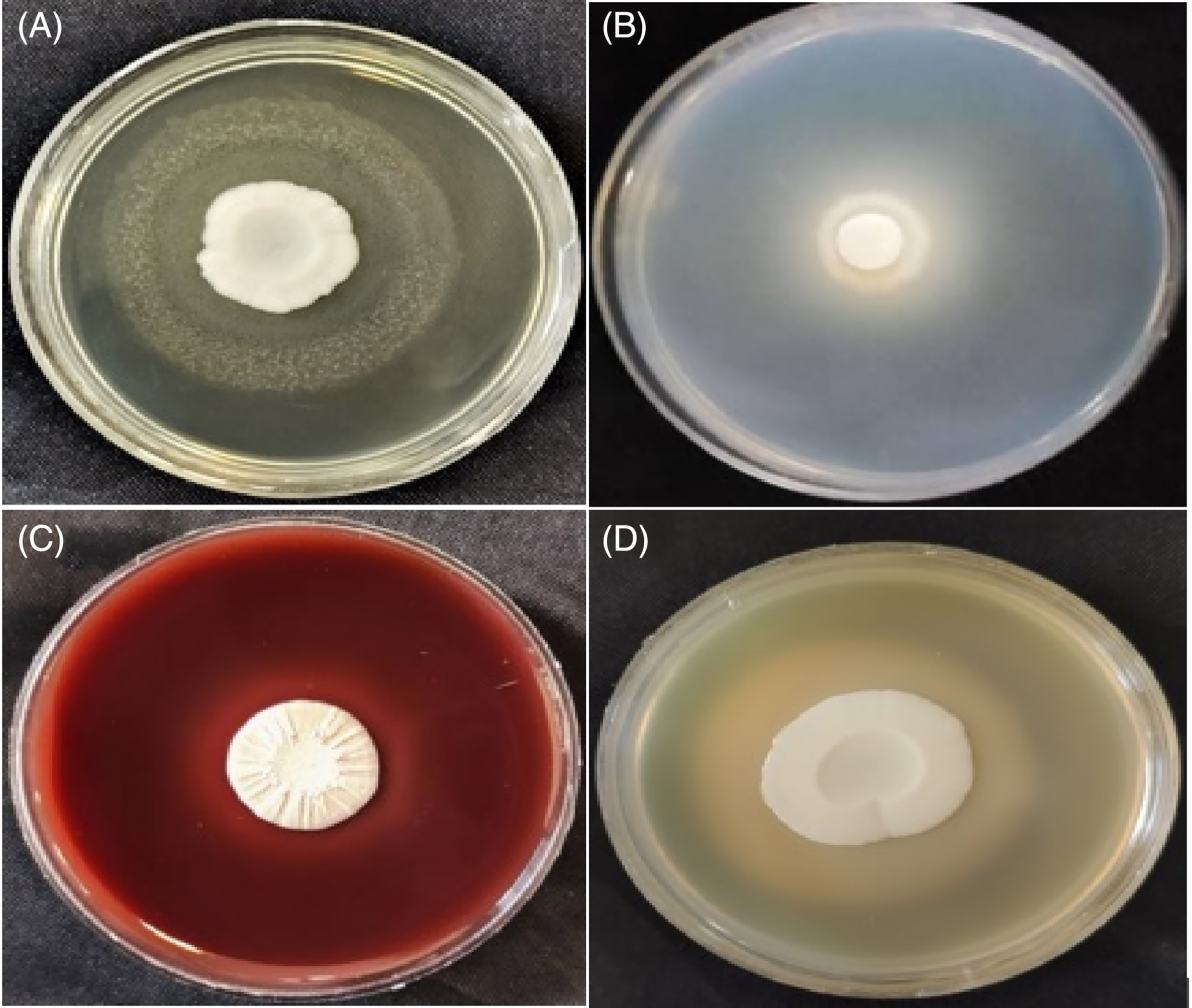
Positive enzyme production of *Candida kefyr* isolates: (A) Esterase. (B) Proteinase. (C) Haemolysin. (D) Phospholipase.

Proteinase activity was detected in 93.9% of all the isolates. Specifically, samples obtained from hospitals exhibited proteinase activity (100%). Also, 90.3% of isolates derived from normal microbiota and dairy origins demonstrated proteinase activity. Furthermore, haemolysin activity was observed in 62.1% of the tested isolates. The highest proportion of haemolysin production, accounting for 95.5%, was found among the normal microbiota isolates Figure 1.

Esterase activity was detected in 9% of the isolates. Among these esterase‐positive isolates, the highest producers of esterase were from dairy samples, while no esterase activity was observed in the hospital samples.

Caseinase production was detected in 16.6% of the samples. Among these positive samples, the highest levels of caseinase activity (18.2%) were detected in normal microbiota and hospital isolates, while the lowest levels (3.6%) were associated with dairy isolates.

Only 3% of the samples showed phospholipase activity. None of the dairy and hospital isolates exhibited phospholipase activity, while all phospholipase producers belonged to normal microbiota isolates. More details about enzyme activity are shown in Table 1.

**TABLE 1:** Enzymatic activity of *Candida kefyr* species.

	Frequency (*n*)									
Sample type	Haemolysin (*n*)		Esterase (*n*)		Phospholipase (*n*)		Proteinase (*n*)		Caseinase (*n*)	
	Negative	4+	Negative	4+	Negative	4+	Negative	4+	Negative	4+
Clinical	18	4	22	0	22	0	0	22	18	4
Dairy	6	16	18	4	22	0	2	20	19	3
Normal microbiota	1	21	20	2	20	2	2	20	18	4
Total	25	41	60	6	64	2	4	62	55	11

### Biofilm formation

The distribution of *C. kefyr* species, based on their sources and biofilm formation intensity, is presented in Table 2. Out of 66 samples, biofilm production was observed in 54 samples (81.8%). There was a variation in the intensity of the biofilm production among the positive‐biofilm producers. Specifically, 24 isolates (36.3%) were intense biofilm producers, 17 isolates (25.7%) were moderate and 13 isolates (19.6%) exhibited a weak positive reaction.

**TABLE 2:** Frequency of Biofilm formation among the isolates.

Sample type	Biofilm formation				
	Negative	Mild	Moderate	Intense	Total
Clinical	7 (31.8%)	5 (22.7%)	5 (22.7%)	5 (22.7%)	22
Dairy	3 (13.5%)	1 (4.5%)	5 (22.7%)	13 (59.1%)	22
Normal microbiota	2 (9.1%)	7 (31.8%)	7 (31.8%)	6 (27.3%)	22

### Relationship between enzyme activities and the sources of isolates

The results of this study indicated that there was no statistically significant difference in the activity levels of esterase, phospholipase, proteinase and caseinase enzymes, as well as in biofilm formation, when comparing clinical isolates with normal microbiota, as evidenced by *p*‐values of 0.488, 0.488, 0.488, 1 and 0.212, respectively. Conversely, a significant difference was observed in the haemolysin activity between clinical isolates and normal microbiota isolates (*p*‐value = 0.001). The results showed that no statistically significant difference was observed between clinical and dairy isolates in esterase, phospholipase, proteinase and caseinase enzyme activity (*p* value = 0.108, 1, 0.488, 1, respectively). Notably, there was a significant difference in haemolysin activity and biofilm formation between clinical and dairy isolates (*p* values = 0.001 and 0.012).

## DISCUSSION

*C. kefyr* is an infrequently encountered yeast species; yet, it remains important in clinical contexts due to its association with invasive infections and its ability to display virulence factors and antimicrobial resistance traits. Studies have shown that *C. kefyr* can exhibit reduced susceptibility to antifungal agents (Ahmad et al., 2020; Nagy et al., 2018). Generally, *C. kefyr* is less pathogenic compared with other *Candida* species, especially *C. albicans*. Therefore, the number of cases isolated and reported from clinical samples has consistently attributed a lower percentage to this species (Jyothi et al., 2021). This is often related to their lower capacity in terms of producing effective enzymes in their pathogenicity. It is essential to note that the enzymatic activity of *C. kefyr* can be influenced by various factors, including environmental conditions such as pH, temperature, and nutrient availability (Emam et al., 2012). Furthermore, the expression and regulation of these enzymes may vary among different strains of *C. kefyr*. However, *C. kefyr* can produce some enzymes with varying intensities (Kantarcioǧlu & Yücel, 2002).

Proteinase activity plays a role in the degradation of proteins, which can facilitate nutrient acquisition and contribute to the pathogenesis of infections. In a study conducted by Sachin et al. (2012) in India, they investigated the proteinase activity of *Candida* species isolated from clinical specimens. The findings revealed that 16.6% of *C. kefyr* isolates exhibited proteinase activity. Conversely, in a separate study by Shirkhani et al. (2016) in Iran, it was shown that all *C. kefyr* isolates (100%) produced the proteinase enzyme. In our study, proteinase activity was observed in a significant majority (93.9%) of the isolates, with all clinical samples exhibiting this enzymatic activity. This suggests that proteinase production may be a common trait among *C. kefyr* strains from clinical sources, potentially facilitating tissue invasion and disease progression.

Haemolysin activity contributes to the pathogenicity of *Candida* species by causing the lysis of red blood cells, which is indicative of damage to host tissues (Asadzadeh et al., 2023). In the research carried out by Yigit et al. (2011) in Turkey, it was demonstrated that 100% of *C. kefyr* had haemolysin activity. In our study, haemolysin activity was detected in 62.1% of the isolates, with a higher prevalence among normal microbiota isolates (95.5%). This finding suggests that *C. kefyr* strains from non‐clinical sources may possess a greater haemolytic potential, which could be important for their survival in diverse environments.

Several issues could explain the increased haemolysin activity observed in *C. kefyr* normal microbiota isolates compared with clinical isolates. First, differential expression of virulence factors may play a crucial role, with normal microbiota isolates potentially upregulating haemolysins to compete with coexisting microorganisms, while clinical isolates might downregulate these factors to evade the host immune response or employ different infection strategies. Environmental adaptation suggests that normal microbiota isolates enhance haemolysin activity to gain advantages in non‐host environments, contrasting with clinical isolates that may select alternative survival mechanisms within the host. Also, clinical isolates may evade host immunity by reducing haemolysin activity to avoid triggering strong immune responses and adopting more subtle infection strategies.

Esterase enzymes play essential roles in lipid metabolism and cellular processes in various organisms. In *Candida* species, esterases may contribute to the breakdown of ester compounds. Mohammed et al. (2019) conducted a study in Greece in 2019 on the stimulation of esterase activity in various *Candida* species, and the findings showed that none of the *C. kefyr* isolates had esterase activity.

In our study, esterase activity was relatively low (9%) and was primarily associated with dairy samples, suggesting a potential role for this enzyme in the colonisation of the gastrointestinal tract, particularly through the consumption of dairy products.

Caseinase hydrolyzes peptide bonds present in casein, and *C. kefyr* is capable of producing this enzyme. This enzymatic activity enables *C. kefyr* to access amino acids derived from casein and allows for the breakdown and utilisation of protein sources present in milk and dairy‐based products. This capability supports its growth and survival in dairy‐rich environments. Therefore, it is expected that this *C. kefyr*, given its inherent nature, exhibits higher levels of protease and caseinase activities. Consequently, considering the availability of these substances in dairy products, environmentally isolated strains should demonstrate more pronounced enzymatic activity. Conversely, clinical isolates are anticipated to exhibit lower caseinase activity compared to environmental strains, although this was not observed in the results. Caseinase production was observed in 16.6% of the isolates, with the highest proportion in normal microbiota and hospital samples. This enzymatic activity may contribute to tissue damage and disease progression in infected individuals (Bow et al., 2010).

Phospholipase is also considered a virulence factor in *Candida* species, including *C. kefyr*. Phospholipase activity is associated with tissue invasion and damage during infection. In our study, phospholipase activity was limited to 3% of the isolates, predominantly in normal microbiota samples. The low prevalence of phospholipase activity among clinical isolates may indicate that this enzyme is not a major contributor to *C. kefyr* pathogenicity in clinical settings. According to Sarkar and Samant (2021), their investigation revealed phospholipase activity in clinical *isolates belonging* to multiple *Candida* species, including *C. kefyr*. Out of the analysed isolates, 12.72% were classified as *C. kefyr* and showed 60% phospholipase activity.

Biofilm production is considered another factor in tissue invasion. Biofilm formation in *Candida* species significantly enhances its pathogenicity by increasing virulence, disrupting host immune responses, and promoting antifungal resistance. Şeker and Özenç (2011) in Turkey in 2011 conducted a study on 8 *C. kefyr* isolates, finding that 62.5% of isolates could produce biofilms. In another study, Zaragoza et al. (2021) reported in 2021 that 73.4% of *C. kefyr* isolates could produce biofilms. In our study, the presence of biofilm formation among 81.8% of the isolates highlights the potential for this species to colonise and adhere to surfaces, contributing to its pathogenicity and resistance to antifungal treatment.

The lack of statistically significant differences in the activity levels of esterase, phospholipase, proteinase, and caseinase enzymes, as well as in biofilm formation, indicates that *C. kefyr's* capacity to produce these enzymes and biofilms may be inherent to the species, regardless of their isolation source. This suggests that both clinical and normal microbiota isolates exhibit similar enzymatic profiles and biofilm‐forming abilities, essential for their survival and establishment in diverse environments. These findings propose that these enzymatic activities and biofilm formation may not serve as primary virulence factors distinguishing pathogenic from non‐pathogenic isolates within this species. Instead, these capabilities likely play crucial roles in fundamental physiological functions, such as nutrient acquisition, rather than directly contributing to pathogenicity.

Understanding the prevalence, antifungal susceptibility profiles, and virulence factors of *C. kefyr* is essential for the effective management and treatment of infections caused by this yeast species. Further research into the epidemiology and mechanisms of resistance of *C. kefyr* can help guide therapeutic strategies and improve patient outcomes in cases of candidemia and other infections. The study delves into the enzymatic characteristics of *C. kefyr*, a species of growing concern in healthcare settings.

## CONCLUSION

The research revealed that none of the clinical isolates displayed esterase activity, yet they showed significant proteinase activity. Furthermore, most isolates from each group tested negative for phospholipase and caseinase. In summary, this study provides valuable insights into the enzymatic profiles and potential biofilm formation abilities of clinical and non‐clinical *C. kefyr* isolates. The variations in enzyme activity and biofilm production among different sources of isolates underscore the complexity of *C. kefyr*'s pathogenic potential. These findings contribute to a better understanding of candidiasis pathogenesis and may guide the development of targeted therapeutic approaches to combat infections caused by this emerging pathogen.

## AUTHOR CONTRIBUTIONS

**Hasti Nouraei:** Writing – original draft; writing – review and editing; investigation; formal analysis. **Samira Zare:** Methodology. **Maryam Nemati:** Methodology. **Neda Amirzadeh:** Methodology. **Marjan Motamedi:** Data curation; validation. **Shafigheh Shabanzadeh:** Methodology; writing – original draft. **Kamiar Zomorodian:** Visualization. **Keyvan Pakshir:** Conceptualization; methodology; data curation; supervision; project administration; formal analysis; validation; visualization; writing – review and editing; writing – original draft; funding acquisition; investigation.

## CONFLICT OF INTEREST STATEMENT

The authors declare no conflict of interest.

## ETHICS STATEMENT

This study was approved by the ethics committee of Shiraz University (IR.SUMS.REC.1401.380 and IR.SUMS.REC.1401.418).

## Data Availability

The data that support the findings of this study are available on request from the corresponding author. The data are not publicly available due to privacy or ethical restrictions.
